# Functional Microbiota for Polypeptide Degradation during Hypertonic Moromi-Fermentation of Pixian Broad Bean Paste

**DOI:** 10.3390/foods9070930

**Published:** 2020-07-14

**Authors:** Lijie Zhang, Yida Bao, Haifeng Chen, Jiaquan Huang, Yan Xu

**Affiliations:** 1 Key Laboratory of Industrial Biotechnology of Ministry of Education, State Key Laboratory of Food Science and Technology, School of Biotechnology, Jiangnan University, Wuxi 214100, China; zhanglj@jiangnan.edu.cn (L.Z.); yidabaojnu@foxmail.com (Y.B.); 2 Sichuan Pixian Douban Company Limited, Chengdu 611730, China; chenhaifeng1991@foxmail.com (H.C.); jiaquanhuang2020@163.com (J.H.)

**Keywords:** polypeptide degradation, functional microbiota, hypertonic condition, Candida, Aspergillus

## Abstract

Traditional fermented bean pastes are indispensable seasonings in many East Asian countries. They are produced via hypertonic solutions by spontaneous fermentation. Functional, unknown microbiota carry great risks for food safety and stable quality. Thus, analysis and subsequent utilization of functional microbiota will be a good strategy to resolve these problems. During bean fermentation, the microbial functions were divided into two stages, including first stage-raw material (polypeptide) degradation and second stage-amino acid catabolism. In this study, we aimed to analyze the functional microbiota of first stage. Omics-studies, including high-throughput sequencing, correlation analysis and extracellular proteome, were used to generate candidate functional microbes for polypeptide degradation in this study. Then, we cultured the candidate functional microbes. After the batch fermentation and enzymatic analysis, we found three strains secreted peptidase and resulted amino acid accumulation, involving *Aspergillus niger*, *Candida zeylanoides* and *Bacillus licheniformis*. Thus, *A. niger*, *C. zeylanoides* and *B. licheniformis* conducted the functional microbiota for polypeptide degrading during hypertonic moromi fermentation. This study supplies a strategy for functional microbiota analysis. In addition, this is the first report that *C. zeylanoides* can secrete proteome and produce amino acids from polypeptide.

## 1. Introduction

Traditional fermented bean pastes, including doenjang, broad bean paste, kimchi and soy sauce, are world-famous seasonings with characteristic flavors, nutrients and healthy ingredients [1,2,3,4,5]. These bean pastes are produced by koji fermentation (a single Aspergillus) and moromi fermentation (spontaneous fermentation under hypertonic condition) [6]. Functionally unknown microbiota in moromi fermentation is the risk factor for stable and reliable quality products. Thus, the analysis and the subsequent utilization of functional microbiota are urgently needed for quality control [7,8]. Till now, the microbial community structure and succession during moromi fermentation have been studied clearly [5], yet the knowledge about functional partitioning and related functional microbiota is limited.

**Figure 1:**
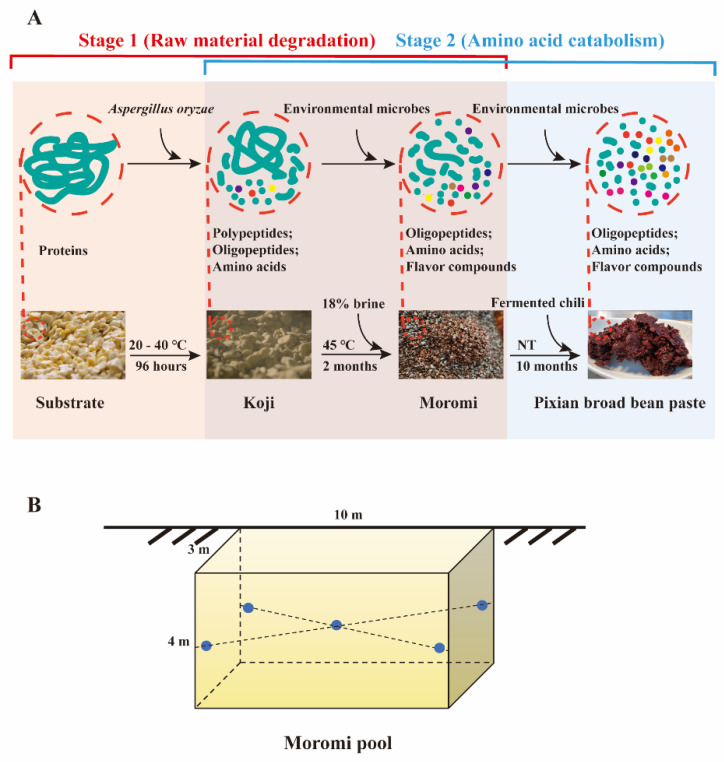
The production of Pixian broad bean paste, including (**A**) the production process and (**B**) the fermentative pool. Protein, as the raw material, can be biodegraded into polypeptides, oligopeptides and amino acids (regarded as microbial function-stage I in this study). Amino acids can then be catabolized into small molecular flavor metabolites (regarded as microbial function-stage II in this study). NT: natural temperature; moromi sample: five points (1.5 m depth) were drawn and mixed.

During the fermentative process of bean paste, protein (as raw material) can be biodegraded into polypeptides, oligopeptides and amino acids [9]. Most amino acids are valuable flavor compounds and nutrients in bean pastes [10]. In addition, as the precursors, amino acids can be catabolized to form small molecular functional ingredients (or their precursors) [11]. Thus, the concentration of amino acids is usually regarded as the quality indicator for bean pastes. In addition, we can partition the microbial functions in the fermentation process into two stages, including “stage I: raw material (protein and polypeptide) degradation to amino acids” and “stage II: amino acids catabolism to small molecular flavor components” (Figure 1A). *Aspergillus oryzae* is known as the functional microbe for raw material degradation in koji fermentation [12]. Limited are studied about whether and which microbes can degrade raw material during hypertonic moromi fermentation.

This study found the phenomena of polypeptide degradation and amino acid accumulation during the late-phase of moromi fermentation of Pixian broad bean paste. In fact, besides Pixian broad bean paste [13,14], similar amino acid accumulation phenomena were also found in the moromi stage of other fermented foods, including soy sauce [15,16], Chinese gray sufu [17] and fish sauce [18]. Meanwhile, a few bacteria were found that can degrade polypeptides under hypertonic condition [5,19]. However, the microbial community structure in moromi is complex, and the functional microbiota associated with amino acid formation remain unknown under the hypertonic moromi condition [20].

In this study, multi-omic studies, including high-throughput sequencing, correlation analysis and extracellular proteome analysis, were conducted to explore the potential functional microbiota for polypeptide degradation. After strain screening, batch-fermentation and enzymatic analysis, we found that three microbes have the capability of secreting peptidase and constructed the functional microbiota for polypeptide degradation during moromi fermentation. This study supplied the microbial functional partition and provided a strategy for functional microbiota analysis during hypertonic moromi fermentation.

## 2. Materials and Methods

### 2.1. Sample Collection

Samples were collected from Sichuan Pixian douban Co., Ltd. (Sichuan, China) at different fermentation times (1, 7, 15, 21, 29, 39, 49 and 60 days) of moromi from October to December 2018. Samples were withdrawn at depths of 1.5 m from moromi pool (10 m length × 3 m width × 4 m depth) by using sample collectors, and each sample was the mixture of 5 points (approximately 200 g each point) (Figure 1B). All samples were stored at −20 °C for further DNA extraction and physicochemical parameter determination. Experiments were conducted in triplicates.

### 2.2. DNA Extraction and Amplification

A sample (7.00 g) was used for genomic DNA extraction by using the E.Z.N.A.^®^ soil DNA Kit (Omega Bio-tek, Norcross, GA, USA) according to kit’s instructions [21]. Then V3–V4 region of the bacterial 16S ribosomal RNA (rRNA) gene was amplified with the universal primers 338F (5′-ACTCCTACGGGAGGCAGCAG-3′) and 806R (5′-GACTACHVGGGTWTCTAAT-3′) [22]. For fungi, the internal transcribed spacer 2 (ITS2) region was amplified with the primers of ITS2 (5′-GCTGCGTTCTTCATCGATGC-3′) and ITS3 (5′-GCATCGATGAAGAACGCAGC-3′) [23]. Barcodes were added in these primers to distinguish each sample. PCR reactions were performed in Beijing Auwigene Tech. Ltd. (Beijing, China). Each PCR system (25 μL volume) contained 10 × Pyrobest buffer (TransGen Biotech, Beijing, China), 2.5 μL; 2.5 mM dNTPs, 2 μL; each primer (10 μM), 1 μL; 0.4 U TransStart fastpfu DNA polymerase; 15 ng template DNA; and double-distilled water (ddH_2_O) (up to 25 μL). Amplification was performed by using the previously described method [24]. Amplicons were posted to Beijing Auwigene Tech. Ltd. (Beijing, China) for high-throughput sequencing by using Miseq Benchtop Sequencer (Illumina, San Diego, CA, USA) for 2 × 300 bp pair-end sequencing [24].

### 2.3. High-Throughput Sequencing Analysis

All the raw Miseq-generated sequences were processed via QIIME (V.1.8.0) [25]. Briefly, high-quality sequences were generated by removing sequences with ambiguous bases > 2, homopolymers > 10, primer mismatches, average quality scores < 20 and lengths (excluding the primer or barcode region) < 120 bp [21]. We used Flash and Pear software to splice the pair-end sequences according to the overlap relationship of the paired-end (PE). Additionally, the minimum overlap was set to 10 bp; the mismatch rate was set to 0.1. Then Fasta sequence was obtained and uploaded to DDBJ (DNA Data Bank of Japan) database with the accession numbers of DRA009305 (ITS) and DRA009313 (16S). Chimera sequences were removed by using the Uchime software (V. 4.1) [26]. After that, operational taxonomic units (OTUs) were clustered using a 97% identity threshold by Qiime’s uclust pipeline [27].

### 2.4. Quantitative Real-Time PCR (qPCR)

The quantifications of fungi and bacteria in moromi fermentation samples were determined by qPCR. Genomic DNA extracted from moromi samples were used as the templates. The V3–V4 region of the 16S rRNA bacterial gene was amplified by using the primers Eub338 (5′-ACTCCTACGGGA GGCAGCAG-3′) and Eub518 (5′-ATTACCGCGGCTGCTGG-3′) [28]. For fungi, the ITS2 region was amplified by using the primers of ITS1 f (5′-TCCGTAGGTGAACCTGCGG-3′) and 5.8 S (5′-CGCTGCGTTCTTCATCG-3′) [28]. The qPCR was performed by using the QuantiFast SYBR green PCR kit commercial kit (Vazyme Biotech Co. Ltd., Nanjing, China) according to our previous protocol [29]. Standard curves of bacterial and fungal biomasses in moromi were created through plotting the C_T_ values of different concentrations of *Bacillus pumilus*’s 16S rRNA gene and *Candida zeylanoides*’s ITS gene. The StepOnePlus instrument (Applied Biosystems, Foster, CA, USA) was used for the qPCR experiment [24,30].

### 2.5. Organic Acid, Glucose and Ethanol Analysis

To analyze organic acid, glucose and ethanol contents in fermented moromi samples, 5 g samples were added to 50 mL centrifuge tubes which contained 20 mL distilled water each. Then the solutions were ultrasonically treated at 0 °C for 30 min. The supernatants were removed and were mixed with 100 g/L trichloroacetic acid (TCA, *v*/*v* 1: 1, Sinopharm, Shanghai, China). Mixtures were centrifuged at 8000× *g* for 5 min under 4 °C. The obtained supernatant was filtered through a 0.22 μm syringe filter (Nylon Acrodisc, Waters Co., Milford, MA, USA) before the quantifications of organic acid, glucose and ethanol contents.

The organic acid, glucose and ethanol contents were determined with high-performance liquid chromatography (Agilent 1260, Santa Clara, CA) (HPLC) with a column Aminex HPX-87H (Bio-Rad). A refractive index detector (RID) and a variable wavelength detector (VWD) (210 nm) were used based on a previous study [31].

### 2.6. pH, Peptides, Amino Acids and Amino Nitrogen Analysis

The concentration of amino acids was determined as in previous reports [32,33]. In brief, moromi samples were diluted several fold before the quantification of amino acids. Then the diluents were centrifuged at 10,000× *g* for 10 min. The supernatant was mixed with 100 g/L trichloroacetic acid (TCA, *v*/*v* 1: 1) and kept at 4 °C for 30 min. The mixture was then centrifuged at 18,000× *g* for 2 min, and the supernatant was passed through a 0.22 μm membrane filter (Nylon Acrodisc, Waters Co., Milford, MA, USA). The free amino acid concentration was determined by HPLC (Agilent 1260, Santa Clara, CA, USA) equipment with the Diamonsil C18 (250 × 4.6 mm 5 μm) column and the variable wavelength detector (VWD) detector. Samples will be derived by o-phthalaldehyde-9-fluorenylmethyl chloroformate (OPA-FMOC). The aqueous phase A was 27.6 mmol/L sodium acetate-triethylamine-tetrahydrofuran (*v*/*v* = 50: 0.11: 2.5), and we adjusted the pH to 7.2 by using acetic acid. Organic phase B was 80.9 mmol/L sodium acetate-methanol-acetonitrile (*v*/*v* =1: 2: 2) and we adjusted the pH to 7.2 by using acetic acid. The detective wavelength and flow rate were 338 nm and 1 mL/min, respectively. Column temperature was 40 °C. Injection volume was 10 μL.

The relative concentrations of polypeptides (1.4–12 kDa) and oligopeptides (≤1.4 kDa) were determined as inthe previous report with slight modifications [34]. In brief, an Agilent 1260 HPLC system (Agilent Technologies, Santa Clara, CA, USA) was used for peptide semi-quantification which was equipped with a TSK gel G2000 column (SWXL 7.8 × 300 mm, Tosoh, Tokyo, Japan) and a VWD detector [35]. The mobile phase was determined as acetonitrile:water = 50:50 (*v*/*v*) with a flow rate of 1 mL/min. The detection wavelength and detection temperature were 215 nm and 25 °C, respectively. A molecular weight standard curve was prepared by using the following standards: tripeptide PHP (Mr. 485), bacitracin (Mr. 1450), aprotinin (Mr. 6500) and cytochrome C (Mr. 12500) (Sigma Chemical Co., St. Louis, MO, USA).

pH was measured by a digital pH meter (FE20 pH Meters, Mettler Toledo Instruments (Shanghai) Co. Ltd., Shanghai, China) according to the user’s manual. Formol titration was used to quantify amino nitrogen, as in previous research [36].

### 2.7. Correlation Analysis among Microbes and Amino Acid Concentration

To analyze the correlation between microbes and chemical profiles (especially amino acids), we calculated all possible Spearman’s rank correlations between the abundant genera (with average abundance top 20) and metabolites using SPSS software (version 24.0). Only correlations index of *p* < 0.05 were considered as valid correlations. We created a network by using Gephi (Web Atlas, Paris, France) to visualize these valid correlations [21]. A *t*-test was carried out to examine the statistical importance of coefficients.

### 2.8. Isolation and Characterization of Salt-Tolerant Strains

LB agar medium with 15% NaCl and 150 ng/mL nystatin was used for the screening of salt-tolerant bacteria at 37 °C. Yeast extract-peptone-dextrose (YPD) agar medium with 15% NaCl and 150 ng/mL cefotaxime sodium (Sangon Biotech, Shanghai, China) was used for the screening of salt-tolerant yeasts at 30 °C. Solid rose bengal medium (R8200, Solarbio, China) with 15% NaCl and 150 ng/mL cefotaxime sodium was used for the screening of salt-tolerant molds at 30 °C. Pixian broad bean paste samples were diluted several times and the diluents were spread on the agar media. Single colonies, including 123 bacteria, 39 yeasts and 22 molds were picked (with different morphology) for purification and identification. Genomes of all single colonies were extracted. 16S rRNA and ITS sequences were cloned by using the bacterial genomes and fungal genomes as templates, respectively. Then strain identification was partly completed by house-keeping gene (16S rRNA or ITS sequence) alignment.

### 2.9. Extracellular Proteomic Analysis

To identify the protease (or peptidase) in moromi samples of Pixian broad bean paste, the global label-free quantitative proteomics analysis was performed (29th day, three biological replicates). A moromi sample of five grams was diluted 5 fold by using double-distilled water (ddH_2_O). Then the diluent was centrifuged 4000× *g* for 5 min at 4 °C. The supernatant was filtered through a 0.22 μm syringe filter (Nylon Acrodisc, Waters Co., Milford, MA, USA) before proteome analysis.

Proteome analysis was carried out at Shanghai Majorbio Bio-pharm Technology Co., Ltd. (Shanghai, China) according to the previous study [37]. In short, enzymes in samples were extracted and digested by trypsin. Then samples were desalted by using Sep-Pak and then be vacuum dried. Peptide concentrations were determined by the peptide quantification kit (Thermo Fisher Scientific, Waltham, MA, USA, catalogue number 23275). The analysis was performed by LC-MS/MS which equipped with an Easy-nLC1200 and a Q-Exactive mass spectrometer.

The sample was loaded into a C18-reversed phase column (75 μm × 25 cm, Thermo Fisher Scientific, Waltham, MA, USA) in buffer A (2% acetonitrile and 0.1% Formic acid) and separated with a linear gradient of buffer B (80% acetonitrile and 0.1% Formic acid) at a flow rate of 300 nL/min with 2 μL of injection volume. For mass spectrometry, survey full-scan MS spectra (*m*/*z* 350–1300) were acquired with a mass resolution of 70 K, followed by 20 sequential high energy collisional dissociation (HCD) MS/MS scans with a resolution of 17.5 K. In all cases, one microscan was recorded using dynamic exclusion of 18 s.

MS/MS spectra were blasted by using Peaks Studio 8.5 software coupled with Bacteria_Fungus-all.uniprot.nr.fasta database and the decoy database. The highest score for a given peptide mass (best match to that predicted in the database) was used to identify parent proteins. The mass spectrometry proteomics data have been deposited to the ProteomeXchange Consortium (http://proteomecentral.proteomexchange.org) via the iProX partner repository [38] with the dataset identifier of PXD016540.

### 2.10. Batch Fermentation to Evaluate the Microbial Function of Polypeptide Degradation

Batch fermentation was carried out to verify whether the potential strains have the capability of degrading polypeptide. The strain used for koji production was *Aspergillus oryzae* HN 3.042 (also named as *A. oryzae* CGMCC 3.951, which was purchased from China General Microbiological Culture Collection Center). Additionally, the koji was then prepared as in an industrial operation [39]. Meanwhile, seed microbes were prepared as follows: bacteria were cultured in LB liquid medium at 37 °C, 200 rpm for 12 h. Yeast were cultured in YPD liquid medium at 30 °C, 200 rpm for 48 h. Mold was cultured in PDA (potato dextrose agar, Solarbio, Beijing, China) solid medium at 28 °C for 72 h. Then, bacteria or yeast cells were washed by normal saline and resuspended to 1 × 10^9^ CFU/mL in 18% sodium chloride (Sinopharm, Shanghai, China), generating seed solutions of bacteria or yeast. Spores of mold were collected, washed and resuspended to 1 × 10^9^ spores/mL in 18% sodium chloride, generating fungal seed solution. Then the grown-up koji was autoclaved for three times at 115 °C for 30 min. Batch fermentation was conducted in 250 mL erlenmeyer flask containing 30 g koji, 32.4 mL saline (18% NaCl) and 3.6 mL seed solution. Batch fermentations inoculated by bacteria were cultured at 37 °C, 180 rpm for 7 days. Batch fermentations inoculated by yeast were cultured at 30 °C, 180 rpm for 7 days. Batch fermentations inoculated by molds were cultured at 28 °C, 180 rpm for 7 days. All samples were stored at −20 °C before the amino nitrogen determination. Protease activity was assayed according to previous report [40]. Strains used in this study are shown in Table 1.

## 3. Results

### 3.1. Amino Acids Accumulate Significantly during Hypertonic Moromi Fermentation

Pixian broad bean paste, known as the “soul of Sichuan cuisine,” is a fermented paste that is popular worldwide and has a characteristic aroma and taste. Amino acid concentration is one of the quality indicators for Pixian broad bean paste. As plenty of proteases (or peptidases) can be secreted by *Aspergillus oryzae* during the koji fermentation, researchers previously regarded the koji fermentation as the unique stage to regulate for higher amino acid accumulation. If amino acid can also be accumulated during the hypertonic moromi fermentation, the moromi stage is a new option to control for higher amino acid production.

**Figure 2:**
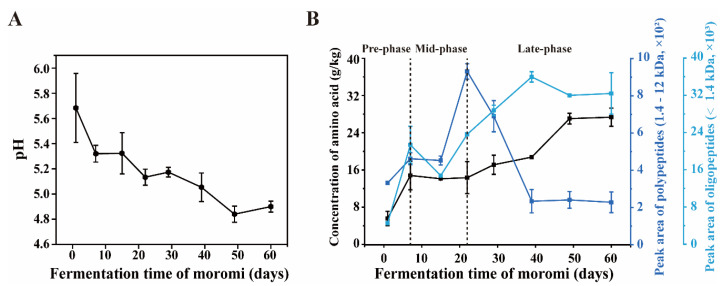
Physicochemical properties during the moromi fermentation. Dynamics of (**A**) pH and (**B**) polypeptide, oligopeptide and amino acid concentrations during the moromi fermentation.

In this study, the physicochemical properties of moromi samples within 60 days were studied. As expected, pH dropped from 5.7 to 4.9 during the fermentation (Figure 2A), which was consistent with a previous report [41]. During the moromi fermentation, concentration of amino acids was determined by amino acid analyzer. Meanwhile, peptides were semi-quantified by HPLC system equipment with a TSK gel G2000 column. As shown in Figure 2B, free amino acid level increased (from 0.57 to 1.49 g/kg) in the first 7 days (pre-phase), and then was stable at 1.4 g/kg from the 7th to 21st fermentation days (mid-phase). It is interesting that a significant accumulation of amino acids appeared (from 1.44 to 2.71 g/kg) at 21st–49th fermentation days (late-phase) during moromi fermentation (Figure 2B, Table 2). Meanwhile, polypeptide (1.4–12 kDa) concentration decreased and the oligopeptide (≤1.4 kDa) concentration increased at 21st–49th fermentation days. Then, we detected the protease activity in moromi samples. At 29 fermentative day, the protease activity at pH 3.0 (acidic) was 2.44 ± 0.22 U/g (moromi sample), and protease activity at pH 7.0 (neutral) was 5.39 ± 0.59 U/g (moromi sample), while the protease activity at pH 10.0 (alkaline) was 9.05 ± 4.80 U/g (moromi sample). These results suggest that there are proteases or peptides playing roles in polypeptide degradation during the late-phase moromi fermentation (21st–49th day).

**Figure 3:**
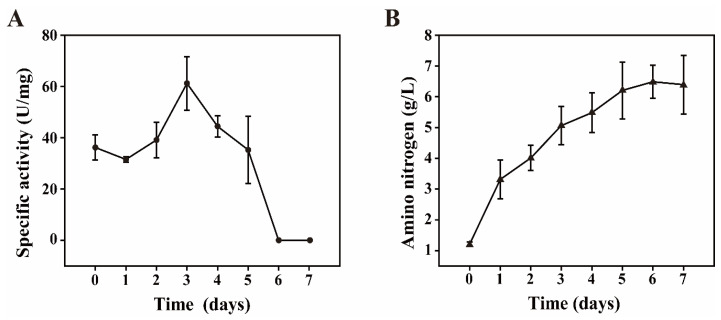
The salt-tolerance of neutral protease excreted from *Aspergillus oryzae*. (**A**) Dynamic curve of specific protease activity in neutral and hypertonic conditions. (**B**) Dynamic curve of amino nitrogen concentration in neutral and hypertonic conditions.

To explore whether the proteases and peptides secreted at the koji stage can also work in the moromi stage, a salt tolerance experiment on the protease of *Aspergillus oryzae* (strain used in koji fermentation) was constructed. The specific activity of the protease was defined as the production of amino acids per minute. We added the mature koji in sterile saline (18% NaCl solution) and then detected the protease activity and amino nitrogen concentration. As shown in Figure 3A,B, protease activity increased in the first 3 days and decreased from the 3rd to the 6th day. Additionally, we could not detect the enzyme activity of protease at 7th day. Meanwhile, concentration of amino nitrogen in saline could not increase on the 7th day. These results suggest that proteases secreted at the koji stage could not contribute to the amino acid accumulation at 21st–49th moromi fermentation days. Thus, there are functional microbiota playing roles in polypeptide degradation during moromi fermentation.

### 3.2. Microbial Community Structure in Moromi Fermentation

Moromi is fermented spontaneously with complex microbial community. To explore what the functional microbiome is for polypeptide degrading, we firstly analyzed the microbial community structure of moromi by using high-throughput sequencing. As shown in Supplementary Materials Table S1, the Good’s coverage of each sample was higher than 97% for both fungi and bacteria and rarefaction curves reached the saturation plateau. These results indicated that the majority of microbial phylotypes in moromi were identified. In all moromi samples, averages of 362 and 108 operational taxonomic units (OTUs, with 97% similarity) were detected for bacteria and fungi, respectively. Proteobacteria and Firmicutes were the dominant bacterial phyla, while Ascomycota and Mucoromycota were the dominant fungal phyla in moromi.

**Figure 4:**
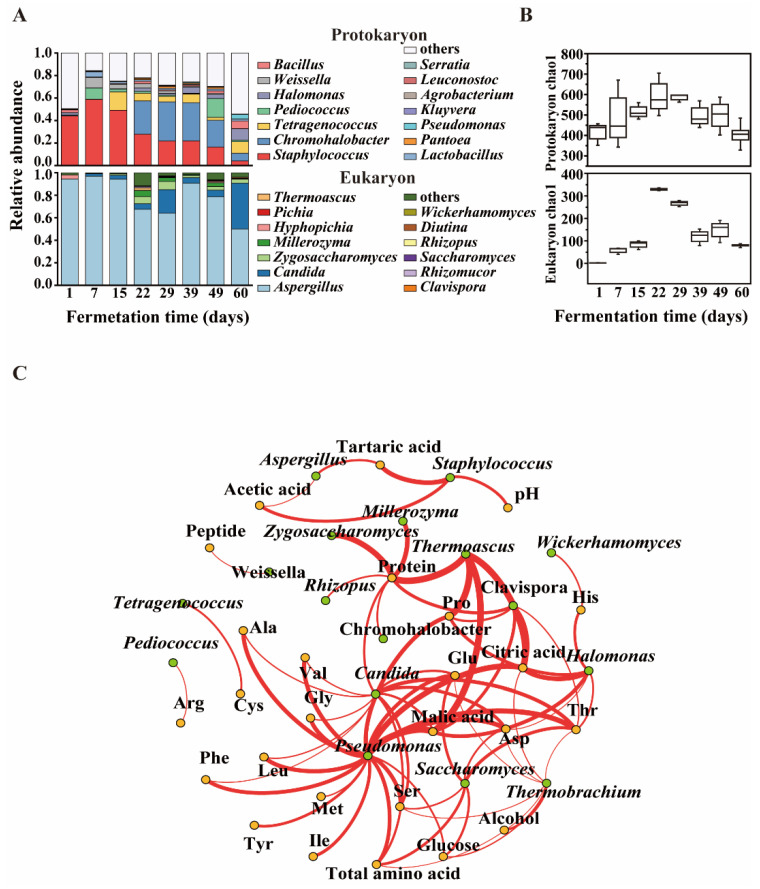
Correlation analysis suggests the potential functional microbiome for amino acid production during hypertonic moromi fermentation. (**A**) The dynamic succession of microbiota in moromi; (**B**) alpha diversity (Chao1) of moromi microbiota; (**C**) Spearman correlation network between the microbes and production of amino acids and organic acids. All edges represent significant correlations (*p* < 0.05). The thickness of a line is highly related to the value of Spearman’s correlation (|*ρ*| > 0.6, *p* < 0.05). Green dots represent microbial genera; Orange dots represent amino acids or organic acids. Red edges mean positive correlation.

Eight bacterial genera were abundant (with over 1% average abundance) during moromi fermentation, including Staphylococcus, Chromohalobacter, Tetragenococcus, Pediococcus, Weissella, Halomonas, Lactobacillus and Bacillus. Genus Staphylococcus was predominant during the 1st–15th fermentation days, while Tetragenococcus, Pediococcus, Weissella and Lactobacillus reached the top at 15th–39th fermentation days, and Chromohalobacter were prevailing in the 15th–60th fermentation days. (Figure 4A). Four fungal genera were abundant (with over 1% average abundance) in the moromi stage, including Aspergillus, Candida, Zygosaccharomyces and Millerozyma. The genus Aspergillus existed in every moromi fermentation process, while Candida dominated on the 60th fermentation day.

The biomass of microbes in moromi was analyzed by using quantitative real-time PCR (qPCR). As shown in Supplementary Figure S1, the biomass of bacteria and fungi decreased continually during the moromi fermentation (bacteria: from (0.96 to 0.02) × 10^8^ copies/mL; fungi: from (0.89 to 0.01) × 10^8^ copies/mL). However, *α* diversity (chao1 index) of bacteria and fungi increased and then decreased during the moromi fermentation, and the highest *α* diversity appeared on the 21st day (Figure 4B). Combined with the phenomenon that polypeptide degrading appeared on the 21st fermentation day, we speculated that there were microbes appearing on the 21st fermentation day that contributed to the polypeptide degradation and amino acid accumulation.

### 3.3. Potential Functional Microbiota for Polypeptide Degrading Was Found by Using Omics Analysis

The complexity of microbiota resulted in the difficulty of functional microbiota analysis for polypeptide degrading. Correlations between the microbial community and metabolite profile were analyzed by using Spearman’s rank correlation coefficient, including 28 genera in moromi and 17 free amino acids, six organic acids, a peptide and a protein. We chose the absolute value of coefficient |*ρ*| above 0.6 and significance *p* less than 0.05 as the nodes of strong correlation. As shown in Figure 4C, positive correlations were observed between 18 genera and 73 metabolite profiles in moromi, while negative correlations were observed between 16 genera and 39 metabolite profiles (Supplementary Materials Table S2).

For fungi, *Candida* showed significantly positive correlations (*p* < 0.05) with the production of eight amino acids, including threonine, serine, alanine, glutamic acid, aspartic acid, isoleucine, leucine and valine. In addition, Saccharomyces was significantly associated with the production of important umami amino acids, including threonine, serine, glutamic acid and aspartic acid. Aspergillus showed a significantly negative correlation with protein concentration, probably because Aspergillus produces protease during the pre-phase of moromi fermentation and plays an important role in protein degrading (Supplementary Table S2). For bacteria, Pseudomonas was significantly associated (*p* < 0.05) with the production of 12 amino acids (threonine, serine, glycine, alanine, glutamic acid, aspartic acid, histidine, isoleucine, leucine, tyrosine, phenylalanine and valine). These results suggest that Candida, Saccharomyces, Aspergillus and Pseudomonas showed positive correlations with the accumulation of amino acids, and might play roles in amino acid production during moromi fermentation.

**Figure 5:**
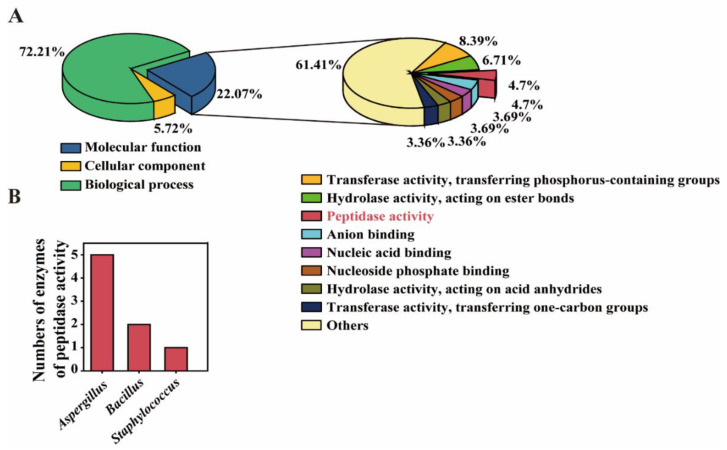
Extracellular proteome analysis by using the moromi sample (fermented at 21st day). (**A**) Data analysis of proteome. (**B**) Number of enzymes that have peptide activity detected by proteome method.

To further analyze the potential functional microbiota for polypeptide degrading, mass spectrometry-based proteomics were evaluated [42]. As the concentration of amino acids increased on the 21st fermentation day (Figure 2B), the proteomic was analyzed by using the 21st day moromi sample. We reserved and analyzed the reads whose −10 logP > 20 and unique peptide > 1. A total of 318 proteins were detected by the extracellular proteomics, among which 22% proteins have a molecular function (annotated by Gene Ontology). Additionally, 4.7% of proteins with molecular functions have peptidase activity (Figure 5A). As shown in Table 3 and Figure 5B, five detected peptidases from proteomics belong to Aspergillus, while two and one detected peptidases belong to Bacillus and Staphylococcus, respectively. Thus, Aspergillus, Bacillus and Staphylococcus were also the potential functional microbes for polypeptide degrading during hypertonic moromi fermentation.

### 3.4. Batch Fermentation and Enzymatic Analysis Were Used to Determine the Functional Microbiota for Polypeptide Degrading

Extracellular proteome and correlation analysis suggested the potential polypeptide degrading microbes, including Aspergillus, Candida, Saccharomyces, Bacillus, Pseudomonas and Staphylococcus. However, knowledge was limited about whether these microbes have active degradative functions on polypeptides. Moreover, *Tetragenococcus halophilus* and *Zygosaccharomyces rouxii* are the significant flavor-producing strains, and have been used in the Japanese soy sauce fermentation industrially. Thus, whether *T. halophilus* and *Z. rouxii* have polypeptide degrading functions was also studied in this study.

**Figure 6:**
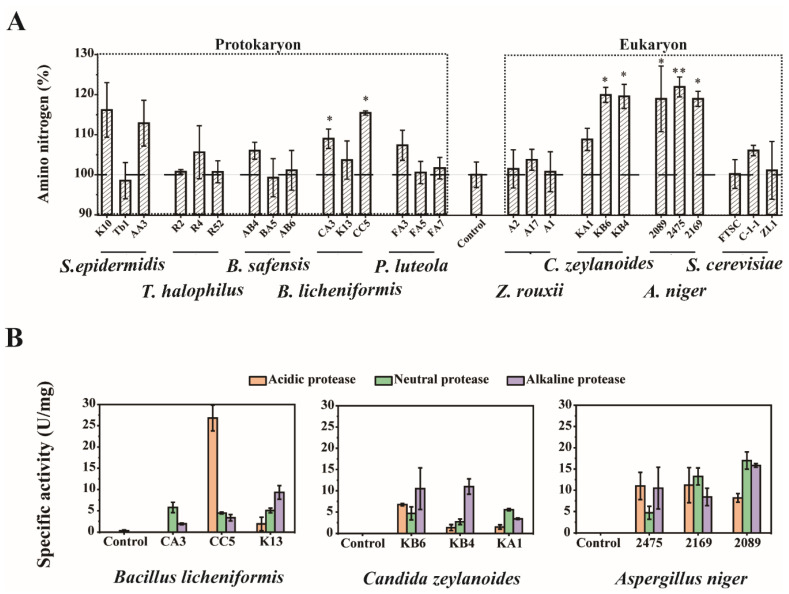
Batch fermentation for detecting the capabilities of polypeptide degrading microbes. (**A**) Concentrations of amino nitrogen in batch fermentation when inoculated with potential polypeptide-degrading microbes. (**B**) Specific activity of protease in acidic (pH 3.0), neutral (pH 7.0) and alkaline (pH 10.0) environments. Every species was represented by three different strains. In Figure 6A, * means *p* < 0.05, while ** means *p* < 0.01.

We screened and identified 184 strains from moromi samples of the 21st day, including 123 bacteria, 39 yeasts and 22 molds (Supplementary Table S3). The most abundant culturable species of Aspergillus, Candida, Saccharomyces, Bacillus, Pseudomonas and Staphylococcus were selected for batch fermentation, including *A. niger, C. zeylanoides, Saccharomyces cerevisiae, Bacillus safensis, B. licheniformis, Pseudomonas luteola* and *Staphylococcus epidermidis*. Additionally, we regarded the moromi sample without inoculation as the control. After 7 days of cultivation, three species, including *B. licheniformis, A. niger* and *C. zeylanoides*, significantly increased the concentration of amino nitrogen (Figure 6A). Then we detected the protease activities of these strains. As shown in Figure 6B, all these strains have protease activity during batch fermentation. These results suggest that *B. licheniformis, A. niger* and *C. zeylanoides* have the capability of producing amino acids by secreting proteases or peptidases, and constructed the functional microbiota for polypeptide degradation during hypertonic moromi fermentation.

## 4. Discussion

This study found that hypertonic moromi fermentation is an important stage for raw material (polypeptide) degradation besides koji. However, the complexity of the microbial community structure in moromi resulted in the difficulty of functional microbiota analysis. In this study, we found a significant accumulation of amino acids during late-phase moromi fermentation. Then, the combination of multi-omics methods (including amplicon sequencing, correlation analysis and extracellular proteome) and batch fermentation were conducted to analyze the functional microbiota for polypeptide degradation during hypertonic moromi fermentation.

Mass spectrometry-based proteomic analysis is a targeted and effective method for localizing polypeptide-degrading functional microbiota when the extracellular protein is recognized as a protease or peptidase. However, some of the genes could not be successfully annotated, suggesting that unidentified peptidases and their hosts could not be recognized by using proteome analysis. Thus, other methods for functional microbiota analysis were needed. Correlation analysis is a widely accepted, un-targeted method for potential function evaluation. Using the correlation analysis, Kim et al., found that Flavobacterium was the key rhizosphere microbe for pathogen resistance [43]. Additionally, Wei et al., found the theabrownin, an active and abundant pigment in Puer tea, could decrease the levels of cholesterol and triglycerides [44]. Thus, the combination of targeted (proteomic analysis) and un-targeted (correlation analysis) methods for functional microbiota analysis might be effective in this study.

As the functional microbe in koji, *Aspergillus oryzae* was popular in raw material (protein and starch) degradation [45,46,47,48]. Diverse proteases and peptidases were secreted in solid cultivation (koji stage) [49]. However, the studies about the function of *Aspergillus* genus during the hypertonic moromi fermentation were limited. This study found that *Aspergillus* genus showed a significant negative correlation with the protein concentration. In addition, *A. niger* can survive and secrete polypeptide-degrading enzymes in hypertonic conditions, potentially including neutral protease 2, subtilisin, carboxypeptidase, aspartic proteinase and aspergillopepsin-1 (Table 3). These results suggest that *Aspergillus* genus can also survive and degrade polypeptides even under the hypertonic condition.

In this study, *Bacillus* showed a positive correlation with the production of amino acids (Supplementary Table S2). Meanwhile, peptidases detected from the moromi sample originated from *Bacillus.* Inoculation of *B. licheniformis* could improve the concentration of amino acids (Figure 6). These phenomena suggested that *B. licheniformis* have the capability of polypeptide degrading, and constructed the functional microbiota in the moromi stage. It is consistent with the previous study that there was high protease activity detected in the *B. licheniformis* culture solution [8]. However, *Bacillus sphaericus* could not improve the amino acid concentration significantly in this study, suggesting that a species-specificity for polypeptide degrading exists in Bacillus genus.

Moromi fermentation contains 18–21% sodium chloride (NaCl). Many halophilic microbes can proliferate in a hypertonic environment [18]. However, as NaCl can reduce the water’s activity and the dissolved oxygen, it is unclear which microbes are active in hypertonic conditions [40,50]. In addition, knowledge is limited about whether the enzymes secreted by functional microbes are stable and whether they play any role in a hypertonic environment [45]. This study newly found the functional microbiota and functional enzymes for polypeptide degradation during hypertonic moromi fermentation.

We failed to detect the accumulation of amino acids in the batch fermentation that was inoculated with *T. halophilus* in this study. This is inconsistent with the previous report that *T. halophilus* can increase amino acid quantities under hypertonic conditions [18]. *T. halophilus* can produce flavor compounds, probably by using amino acids as substrates. Moreover, Li et al., pointed out that *Pseudomonas* was significantly associated with the production of aldehydes, which can be catalyzed from amino acids by transaminase [51]. Thus, during the batch fermentation, *T. halophilus* and Pseudomonas might produce and catabolize amino acids simultaneously, and thus, no obvious accumulation of amino acids was found in this study. Thus, it is worthy to further investigate whether *T. halophilus* or *P. luteola* are capable of polypeptide degradation.

## 5. Conclusions

In this study, we observed that amino acids accumulate while polypeptides decrease during the hypertonic moromi fermentation. Then we studied whether and which microbiota had effects on polypeptide degradation. Multi-omics studies were conducted to investigate the potential microbiota for polypeptide degrading. After the strain isolation, batch fermentation and enzymatic analysis, we found that *A. niger*, *C. zeylanoides* and *B. licheniformis* can improve amino acid concentration and construct the functional microbiota for polypeptide degrading. This is the first report showing that *C. zeylanoides* can secrete peptidase in hypertonic conditions. This study supplies a strategy for functional microbiota analysis, and will be helpful for the industrial control for higher quality and safer fermented bean paste.

## Figures and Tables

**Table 1:** Strains used in this study.

Isolated Strains	Accession Number	LBM ID	Description
*Staphylococcus epidermidis* K10	MN428237.1	LBM 19032	
*S. epidermidis* AA3	CP045648.1	LBM 19054	Isolated from Pixian broad bean paste
*S. epidermidis* Tb1	KM253004.1	LBM 19116	
*Tetragenococcus halophilus* R2	MK063722.1	LBM 19170	
*T. halophilus* R4	MK063724.1	LBM 19172	LBM strain collection
*T. halophilus* R52	GQ150506.1	LBM 19211	(isolated from fermented food)
*Bacillus safensis* AB4	MN704448.1	LBM 19056	
*B. safensis* AB6	MN704552.1	LBM 19057	Isolated from Pixian broad bean paste
*B. safensis* AA5	MN704552.1	LBM 19058	
*Bacillus licheniformis* CA3	KU922147.1	LBM 19067	
*B. licheniformis* CC5	CP045814.1	LBM 19115	Isolated from Pixian broad bean paste
*B. licheniformis* K13	MK414971.1	LBM 19103	
*Pseudomonas luteola* FA3	CP044086.1	LBM 19128	
*P. luteola* FA7	NR_114215.1	LBM 19222	Isolated from Pixian broad bean paste
*P. luteola* FA9	CP044085.1	LBM 19223	
*Zygosaccharomyces rouxii* A2	AB363049.1	LBM 29111	
*Z. rouxii* A17	MH669504.1	LBM 29104	LBM strain collection
			(isolated from fermented food)
*Z. rouxii* A1	MK294302.1	LBM 29101	
*Candida zelanoides* KA1	KX376266.1	LBM 29033	
*C. zelanoides* KB6	MN073455.1	LBM 29038	Isolated from Pixian broad bean paste
*C. zelanoides* KB4	MK613257.1	LBM 29037	
*Aspergillus niger* 2089	MN420840.1	LBM 39001	
*A. niger* 2475	MN535797.1	LBM 39003	LBM strain collection
			(isolated from fermented food)
*A. niger* 2169	MN611114.1	LBM 39002	
*Saccharomyces cerevisiae* FTSC	MN585900.1	LBM 27012	
*S. cerevisiae* C-1-1	MN585905.1	LBM 27046	LBM strain collection
			(isolated from fermented food)
*S. cerevisiae* ZL1	MN158119.1	LBM 27145	
*Aspergillus oryzae* HN 3.042			CGMCC 3.951

**Table 2:** Concentrations of amino acid in moromi samples of Pixian broad bean paste (g/kg) ^a^.

Amino Acid	Fermentation Time (Day)							
	1	7	15	22	29	39	49	60
Threonine	0.20 ± 0.02	0.68 ± 0.16	0.48 ± 0.23	0.71 ± 0.19	0.84 ± 0.10	1.17 ± 0.30	1.30 ± 0.08	1.33 ± 0.12
Serine	0.01 ± 0.01	0.07 ± 0.01	0.09 ± 0.04	0.07 ± 0.02	0.15 ± 0.03	0.21 ± 0.05	0.25 ± 0.09	0.29 ± 0.14
Glycine	0.14 ± 0.03	0.35 ± 0.08	0.56 ± 0.27	0.42 ± 0.11	0.51 ± 0.07	0.72 ± 0.22	0.83 ± 0.07	0.89 ± 0.09
Alanine	0.29 ± 0.04	0.81 ± 0.16	1.17 ± 0.48	0.92 ± 0.22	1.06 ± 0.13	1.52 ± 0.40	1.70 ± 0.11	1.85 ± 0.11
Proline	0.55 ± 0.02	0.50 ± 0.14	0.89 ± 0.43	0.91 ± 0.42	0.81 ± 0.20	1.11 ± 0.30	1.36 ± 0.10	1.29 ± 0.11
Glutamic acid	0.81 ± 0.13	2.64 ± 0.54	2.43 ± 0.05	2.47 ± 0.51	2.95 ± 0.39	3.18 ± 0.08	4.45 ± 0.15	4.50 ± 0.21
Aspartic acid	0.46 ± 0.02	1.15 ± 0.18	1.26 ± 0.03	1.21 ± 0.30	1.76 ± 0.26	2.46 ± 0.66	2.81 ± 0.16	2.91 ± 0.22
Lysine	0.55 ± 0.17	1.80 ± 0.46	2.31 ± 1.19	1.43 ± 0.33	1.53 ± 0.23	2.03 ± 0.69	2.26 ± 0.04	2.33 ± 0.12
Histidine	0.14 ± 0.04	0.05 ± 0.01	0.06 ± 0.03	0.05 ± 0.01	0.06 ± 0.01	0.53 ± 0.64	0.35 ± 0.35	1.01 ± 0.07
Arginine	1.18 ± 0.40	2.47 ± 0.48	2.70 ± 1.06	1.73 ± 0.24	1.73 ± 0.26	2.13 ± 0.40	2.66 ± 0.10	2.62 ± 0.06
Methionine	0.09 ± 0.02	0.20 ± 0.04	0.30 ± 0.14	0.20 ± 0.05	0.25 ± 0.04	0.33 ± 0.11	0.33 ± 0.04	0.30 ± 0.05
Isoleucine	0.23 ± 0.08	0.56 ± 0.14	0.91 ± 0.42	0.68 ± 0.21	0.80 ± 0.11	1.12 ± 0.34	1.30 ± 0.13	1.29 ± 0.15
Leucine	0.38 ± 2.09	1.06 ± 0.23	1.66 ± 0.70	1.23 ± 0.34	1.38 ± 0.17	1.92 ± 0.60	2.29 ± 0.20	2.30 ± 0.28
Tyrosine	0.23 ± 0.12	0.75 ± 0.13	0.92 ± 0.27	0.72 ± 0.15	0.85 ± 0.09	1.16 ± 0.33	1.38 ± 0.09	1.40 ± 0.09
Phenylalanine	0.29 ± 0.09	0.66 ± 0.15	1.02 ± 0.41	0.75 ± 0.22	0.81 ± 0.09	1.09 ± 0.33	1.37 ± 0.15	1.37 ± 0.17
Valine	0.33 ± 0.11	0.79 ± 0.17	1.18 ± 0.51	0.88 ± 0.23	0.98 ± 0.13	1.39 ± 0.42	1.58 ± 0.14	1.63 ± 0.20
Cysteine	0.00 ± 0.00	0.01 ± 0.01	0.02 ± 0.02	0.01 ± 0.01	0.01 ± 0.01	0.02 ± 0.02	0.01 ± 0.01	0.01 ± 0.01
Total	5.41 ± 1.34	14.55 ± 3.09	17.96 ± 6.27	14.39 ± 3.55	16.49 ± 2.32	22.09 ± 5.89	26.24 ± 2.02	27.32 ± 2.18

^a^ Mean ± standard deviation (*n* = 3).

**Table 3:** Peptidases detected by extracellular proteome.

Enzyme	Accession Number	Abundance %	Description	Source
Neutral protease2	sp|B8NWE1|NPIIB_ASPFN	6.96 × 10^−4^	EC:3.4.24.39. metalloendopeptidase activity	*Aspergillus flavus*
Subtilisin-like serine protease	XP_022386201.1	1.50 × 10^−5^	Serine-type endopeptidase activity	*Aspergillus bombycis*
Carboxypeptidase	XP_022388195.1	1.09 × 10^−5^	Serine-type carboxypeptidase activity	*A. bombycis*
Aspartic proteinase	CEL07294.1	4.32 × 10^−5^	Aspartic-type endopeptidase activity	*Aspergillus calidoustus*
Aspergillopepsin-1	sp|B8NLY9|PEPA_ASPFN	1.07 × 10^−3^	EC:3.4.23.18. aspartic-type endopeptidase activity	*A. flavus*
Cyanophycinase	WP_099000026.1	2.04 × 10^−5^	Serine-type peptidase activity	*Bacillus aryabhattai*
CocE/NonD family hydrolase	WP_066152222.1	2.29 × 10^−6^	Dipeptidyl-peptidase activity	*Bacillus krulwichiae*
Methionyl aminopeptidase	WP_103328270.1	2.25 × 10^−4^	EC:3.4.11.18. metalloaminopeptidase activity	*Staphylococcus petrasii*

## Supplementary Materials

The following are available online at https://www.mdpi.com/2304-8158/9/7/930/s1. Figure S1: The bacterial and fungal biomasses in moromi. Table S1: The sequencing reads, OTU numbers and Good’s coverage. Table S2: Spearman correlation coefficient between microbes and metabolites. Table S3: Strain list cultured from moromi sample.
