# Supplementary material for: Functional Microbiota for Polypeptide Degradation during Hypertonic Moromi-Fermentation of Pixian Broad Bean Paste
Source: Foods. 2020 Jul 14;9(7):930. doi: 10.3390/foods9070930 (PMC7404569; doi:10.3390/foods9070930)
Supplement: Supplementary file 1 [file foods-09-00930-s001.zip › Supporting informaiton/Supporting information data sheet 1.docx]

Supplementary Material

Supplementary Data Functional microbiota for polypeptide-degrading during hyperosmotic moromi-fermentation of Pixian broad bean paste

Yida Bao^1^, Lijie Zhang^1^, Haifeng Chen^2^, Jiaquan Huang^2^, Yan Xu^*1^

^1^Key Laboratory of Industrial Biotechnology of Ministry of Education, State Key Laboratory of Food Science and Technology, School of Biotechnology, Jiangnan University, Wuxi, China; ^2^Sichuan Pixian Douban Co., Ltd.

***Correspondence:**Corresponding author
yxu@jiangnan.edu.cn

**This file includes:**

Supplementary Figures (Fig. S1)

**Other supplementary information for this manuscript includes the following:**

Supplementary Table S1 as an Excel file: Supplementary Table S1.xls in data sheet 2

Supplementary Table S2 as an Excel file: Supplementary Table S2.xls in data sheet 2

1. **Supplementary Figures and Tables**

## Supplementary Figures


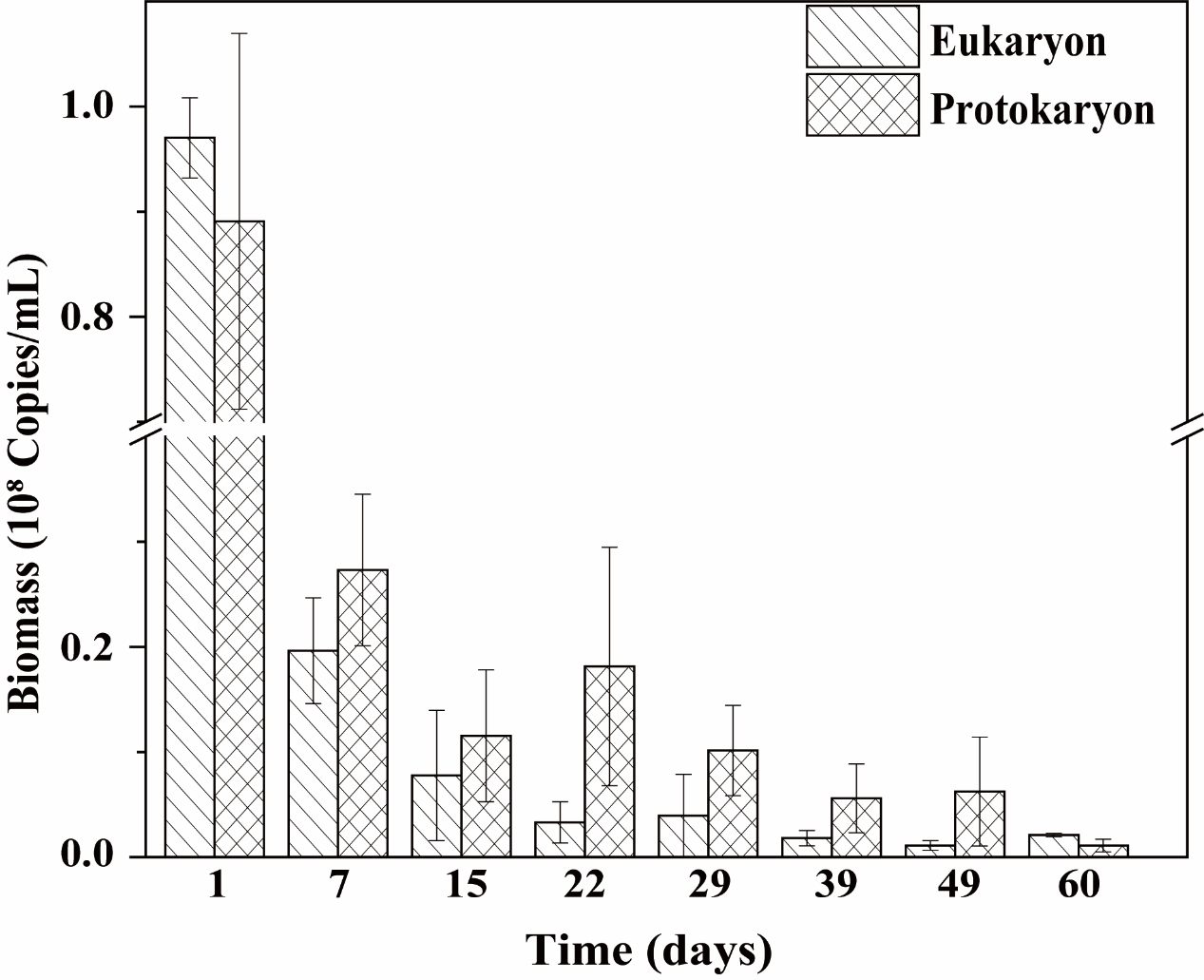


**Supplementary Fig. S1.** The biomass of moromi fermentation using Quantitative real-Time PCR (qPCR). the biomass of bacteria and fungi decreased continually during the moromi fermentation.
